# Local and Regional Scale Heterogeneity Drive Bacterial Community Diversity and Composition in a Polar Desert

**DOI:** 10.3389/fmicb.2018.01928

**Published:** 2018-08-21

**Authors:** Kelli L. Feeser, David J. Van Horn, Heather N. Buelow, Daniel R. Colman, Theresa A. McHugh, Jordan G. Okie, Egbert Schwartz, Cristina D. Takacs-Vesbach

**Affiliations:** ^1^Department of Biology, University of New Mexico, Albuquerque, NM, United States; ^2^Department of Biological Sciences, Colorado Mesa University, Grand Junction, CO, United States; ^3^School of Life Sciences, School of Earth and Space Exploration, Arizona State University, Tempe, AZ, United States; ^4^Department of Biological Sciences, Northern Arizona University, Flagstaff, AZ, United States

**Keywords:** environmental heterogeneity, 16S rRNA genes, gradient analysis, spatial scale, polar desert, McMurdo Dry Valleys

## Abstract

The distribution of organisms in an environment is neither uniform nor random but is instead spatially patterned. The factors that control this patterning are complex and the underlying mechanisms are poorly understood. Soil microbes are critical to ecosystem function but exhibit highly complex distributions and community dynamics due in large part to the scale-dependent effects of environmental heterogeneity. To better understand the impact of environmental heterogeneity on the distribution of soil microbes, we sequenced the 16S rRNA gene from bacterial communities in the microbe-dominated polar desert ecosystem of the McMurdo Dry Valleys (MDV), Antarctica. Significant differences in key edaphic variables and alpha diversity were observed among the three lake basins of the Taylor Valley (Kruskal–Wallis; pH: χ^2^ = 68.89, *P* < 0.001, conductivity: χ^2^ = 35.03, *P* < 0.001, observed species: χ^2^ = 7.98, *P* = 0.019 and inverse Simpson: χ^2^ = 18.52, *P* < 0.001) and each basin supported distinctive microbial communities (ANOSIM *R* = 0.466, *P* = 0.001, random forest ratio of 14.1). However, relationships between community structure and edaphic characteristics were highly variable and contextual, ranging in magnitude and direction across regional, basin, and local scales. Correlations among edaphic factors (pH and soil conductivity) and the relative abundance of specific phyla were most pronounced along local environmental gradients in the Lake Fryxell basin where Acidobacteria, Bacteroidetes, and Proteobacteria declined while Deinococcus–Thermus and Gemmatimonadetes increased with soil conductivity (all *P* < 0.1). Species richness was most strongly related to the soil conductivity gradient present within this study system. We suggest that the relative importance of pH versus soil conductivity in structuring microbial communities is related to the length of edaphic gradients and the spatial scale of sampling. These results highlight the importance of conducting studies over large ranges of key environmental gradients and across multiple spatial scales to assess the influence of environmental heterogeneity on the composition and diversity of microbial communities.

## Introduction

Understanding the controls on the distribution of organisms has been one of the fundamental goals of ecology for the past century. Numerous studies suggest that this distribution is neither uniform nor random, but instead spatially patterned (Legendre and Fortin, 1989; Ettema and Wardle, 2002; O’Brien et al., 2016). However, the factors that control this patterning are complex, multifaceted, and include abiotic characteristics, biotic interactions, and stochastic events. Additionally, many ecological processes that influence the distribution, abundance, and interactions of species are scale-dependent, including the flow of individuals within an environment, the impacts and extent of disturbances, and variation in environmental conditions and habitability (Leibold et al., 2004). Spatial variation in environmental conditions, referred to here as environmental heterogeneity, is often cited as the primary driver of biodiversity (Stein et al., 2014; Coyle and Hurlbert, 2016). Environmental heterogeneity increases resource diversity and provides opportunities for niche partitioning and speciation events (Stein et al., 2014), allowing for increased species coexistence (MacArthur and Levins, 1964; Coyle and Hurlbert, 2016). However, there is an inherent tradeoff between environmental heterogeneity and diversity: as niche opportunities increase, the effective area available for each species decreases and the probability of stochastic extinctions rises (Allouche et al., 2012). Consequently, environmental heterogeneity is an important factor for species coexistence, persistence, and diversification (Stein et al., 2014).

Recent evidence suggests that environmental heterogeneity strongly impacts the distribution of microbial communities. However, these relationships are complex and contingent on several factors. First, there are the confounding effects of spatial scale.

Larger spatial areas generally encompass greater environmental heterogeneity which can come in the form of longer gradient lengths (e.g., wider ranges of conditions) or harsher gradient severity (e.g., more extreme conditions). Thus, as environmental heterogeneity is inextricably linked with spatial scale, patterns observed at small scales do not necessarily correspond to those found at larger scales (Franklin and Mills, 2009; Geyer et al., 2013; Van Horn et al., 2013; Bar-Massada and Wood, 2014; Stein et al., 2014). Additionally, because environmental heterogeneity typically involves simultaneous changes in numerous abiotic and biotic parameters, the impacts of one variable on microbial community structure may become overwhelmed by the impacts of other variables, creating threshold effects and difficulties in resolving drivers of community change. Clades within communities also respond differently to various environmental factors because their diverse physiological adaptations to resource limitations or environmental severity result in differences in relative fitness (Franklin and Mills, 2009; Geyer et al., 2013). Finally, a related complexity is the presence of “contextual effects,” i.e., the observation that relationships between environmental factors and communities depend on geographic context (Van Horn et al., 2013). Therefore, multi-scale analyses along environmental gradients and across various landscape contexts are necessary to understand the abstruse dynamics of scale-dependent ecological processes that structure microbial communities.

A recent review of environmental heterogeneity-diversity studies noted that soil habitats are particularly underrepresented in the literature (Stein et al., 2014), despite the critical importance of soil microbial communities to ecosystem functioning (Cavigelli and Robertson, 2000). This limited understanding is due to the extremely high diversity of these systems, which often contain thousands of microbial species per-gram of soil (Torsvik et al., 1996), the physical and chemical complexity and heterogeneity of soil habitats (Nannipieri et al., 2003), and the frequency of stochastic disturbances which result in the formation of complex spatial patterns (O’Brien et al., 2016). Spatially structured communities have been observed at continental scales (Fierer et al., 2009; Lauber et al., 2009; Barberán et al., 2012; Fierer et al., 2012, 2013) to centimeter scales (Morris, 1999; Grundmann and Debouzie, 2000; Franklin and Mills, 2009; O’Brien et al., 2016), but the underlying ecological mechanisms remain difficult to decipher. Two master variables frequently implicated in controlling microbial diversity and community composition are pH and salinity. Several studies have suggested that the most influential variable on soil microbial community composition is pH (Fierer and Jackson, 2006; Baker et al., 2009; Lauber et al., 2009; Smith et al., 2010) while others have suggested salinity (Lozupone and Knight, 2007; Zeglin et al., 2011; Lee et al., 2012). We propose that differences in scale-dependent environmental heterogeneity may underlie these conflicting conclusions, and we suggest that determining the impacts of environmental gradient lengths and severity are crucial to unlocking this puzzle.

The soils of the McMurdo Dry Valleys (MDV) are an ideal natural laboratory to investigate the relationships between microbial community structure, edaphic characteristics, and scale. Considered the coldest, driest, most oligotrophic desert on Earth (Hopkins et al., 2006; Cary et al., 2010), the MDV are a microbe-dominated ecosystem, as extreme conditions prohibit the existence of higher plants and animals. In the absence of vegetation and biotic interactions such as herbivory, MDV soils are shaped into distinct spatial patterns by physicochemical factors including moisture, salinity, pH, and carbon availability (Barrett et al., 2004; Lee et al., 2012; Van Horn et al., 2013, 2014; Okie et al., 2015). MDV soils contain relatively low levels of biodiversity providing an ideal model system with which to investigate community-environment interactions. Invertebrates are rare and phyla include only the protozoa, rotifers, tardigrades, nematodes and *Collembola*. At many sites, only one species of nematode, the endemic *Scottnema lindsayae* are found (Freckman and Virginia, 1997; Barrett et al., 2004). Fungal and archaeal community distribution is similarly patchy (Arenz and Blanchette, 2011; Richter et al., 2014) and while bacteria are ubiquitous, their diversity is on average, approximately one third of that found in most other soils (Van Horn et al., 2013, 2014).

Previous research has leveraged the existence of patterned ground formations or soil polygons in the MDV and elsewhere to investigate the role of geomorphic history (Brinkmann et al., 2007) and physicochemical variation across multiple spatial scales (Barrett et al., 2004) on soil biodiversity, although the former study was focused on cyanobacteria and the latter on invertebrates. Strong physical and biogeochemical gradients form along soil polygons that are related to nematode abundance and biodiversity variation. However, a similar, systematic investigation of MDV soil bacterial communities has not been conducted, despite their ubiquity across the MDV landscape (Takacs-Vesbach et al., 2010) and that multiple studies have shown that MDV soil bacterial communities are active under *in situ* conditions (Schwartz et al., 2014; Buelow et al., 2016). Understanding the effects of environmental heterogeneity is crucial to predicting the controls on the diversity and distribution of soil microbial communities. However, untangling these relationships is complicated by landscape contexts and spatial-scale dependent evolutionary and ecological mechanisms. Using soil polygons as the organizing framework across multiple biogeochemically diverse basins is an ideal approach to address environmental heterogeneity. The goal of this study was to explore the effects of edaphic pH and electrical conductivity (EC) gradients on bacterial community diversity and composition as they vary across regional, watershed basin, and local scales. Specifically, we examined the degree to which these key edaphic gradients structure microbial communities by disentangling the impacts of (1) spatial scale, (2) edaphic gradients, and (3) related threshold effects on microbial distribution patterns.

## Materials and Methods

### Site, Sampling Description, and Chemical Analysis

The McMurdo Dry Valleys, Victoria Land, Antarctica (77° 30′ S, 163° 00′ E) comprise one of the harshest environments on Earth, with air and surface soil temperatures averaging between -15 and -30°C and extremes ranging from -60 to 25°C on the soil surface (Doran et al., 2002). The MDV are the largest ice-free zone in continental Antarctica (Fountain et al., 1999) with a total area of 22,700 km^2^ and an ice-free area of 4,500 km^2^ (Levy, 2013). The MDV receive little precipitation (<10 cm snow per year; Keys, 1980), most of which is lost through sublimation (Clow et al., 1988). The low soil moisture and precipitation results in an accumulation of salts and high pH in the upper soil stratum (Bockheim, 1997). Physicochemical gradients across the MDV are especially heterogeneous owing to its diverse glacial history (Lee et al., 2012). The hyper-arid mineral soils are primarily categorized as Anhyorthels or Anhyturbels and contain very little organic matter (Bockheim, 1997). Furthermore, the soils of the MDV are subjected to frequent freeze-thaw cycles that create physical sorting of rocks and soil particles and cause the expansion and contraction of permafrost layers 0.2–0.5 m below the ground surface (Kessler et al., 2001; Bockheim, 2002; Kessler and Werner, 2003). These processes create patterned ground formations, termed soil polygons. The polygons are clearly distinguished by intersecting troughs along their margins, and are a prominent landscape feature useful for geometrically scaling local ecological information (Barrett et al., 2004). Within polygons, significant differences in soil chemistry have been detected that are presumably due to naturally occurring edaphic heterogeneity and/or soil polygon mechanics, i.e., cryoturbation (Bockheim, 2002; Barrett et al., 2004).

Soils were aseptically collected from polygons during the austral summer of 2012 from the three major hydrological basins of the Taylor Valley: Bonney, Fryxell, and Hoare. A map of approximate sampling locations and major topographical features is provided in the **Supplementary Figure S1**. Eight soil polygons with an approximate radius of 6 m were randomly selected within each lake basin. Polygons within the Bonney basin were between 380 and 410 m from the lake margin, polygons within the Hoare basin 270–280 m, and polygons within the Fryxell basin were approximately 120 m away. Within each polygon, five samples were aseptically collected with sterilized scoops to a depth of approximately 10 cm along radial transects beginning from the trough edge to the center (at 0, 0.4, 0.8, 2, and 6 m), for a total of 120 samples. Soils were collected into sterile Whirl-Pak bags. Within 24 h, soils for molecular analysis were subsampled into sterile tubes by preserving approximately 10 g of soil with an equal volume of sucrose lysis buffer (Giovannoni et al., 1990). Samples were stored at -20°C until extraction. Soil pH was determined on 1:2 soil/deionized water extracts using an Orion pH probe. EC of 1:5 soil/water extracts was measured with a Yellow Springs Instrument 3100 conductivity meter.

### DNA Extraction, Sequencing, and Sequence Analysis

DNA from 0.7 g of soil was extracted using the cetyltrimethylammonium bromide (CTAB) method (Hall et al., 2008; Mitchell and Takacs-Vesbach, 2008). Barcoded amplicon pyrosequencing of 16S rRNA genes was performed as previously described (Dowd et al., 2008; Van Horn et al., 2013, 2014) using V6 universal bacterial primers 939F 5′ TTG ACG GGG GCC CGC ACA AG-3′ and 1492R 5′-GTT TAC CTT GTT ACG ACT T-3′ on a Roche 454 FLX instrument using Roche titanium reagents following the manufacturer’s instructions.

The 16S rRNA gene sequences were quality filtered, denoised, screened for PCR errors, and chimera checked using default parameters in AmpliconNoise (Quince et al., 2011). The Quantitative Insights into Microbial Ecology (QIIME) pipeline was used to analyze the 16S rRNA gene sequences (Caporaso et al., 2010a). Unique 16S rRNA gene sequences or operational taxonomic units (OTUs) were identified using the 97% DNA identity criterion using UCLUST (Edgar, 2010). A representative sequence was chosen from each OTU and aligned using the PyNAST aligner (Caporaso et al., 2010b) and the Greengenes core set (version 13.8) (DeSantis et al., 2006). Taxonomic assignments of the OTUs were made using the Ribosomal Database Classifier program (Wang et al., 2007).

All measures of community diversity (observed species, inverse Simpson, Good’s coverage, Bray–Curtis, and Jaccard distances) and composition were performed with randomly selected subsets of 500 sequences per sample to standardize for varying sequencing efforts across samples. Raw sequence data from this study are available through the NCBI Sequence Read Archive as PRJNA436435. The individual sff files from this study were assigned the accession numbers SAMN08624939–SAMN08625045.

### Statistical Analysis

The normality of pH, EC, and alpha diversity distributions were assessed using Shapiro–Wilk tests. Significant differences in pH, EC, and alpha diversity data were assessed using non-parametric Kruskal–Wallis rank sum tests followed by *post hoc* pairwise Tukey honest significant difference (HSD) tests, corrected for multiple comparisons.

Data were pooled among all three lake basins for regional scale analysis. Patterns in microbial communities among and within lake basins (basin scale) were analyzed using non-metric multidimensional scaling (NMDS) using Bray–Curtis and Jaccard distances. Differences in microbial community composition (i.e., Bray-Curtis distances) were assessed by an Analysis of Similarity (ANOSIM) test with 999 permutations to assess significance. In addition, we investigated the degree to which microbial community profiles were associated with environmental factors by using the Random Forests classification algorithm (Breiman, 2001) implemented in QIIME’s supervised_learning.py command with 10-fold cross-validation on a rarefied OTU table (-e 500) that was filtered to remove OTUs with less than 10 sequences. Performance of the random forests classifier is reflected by the ratio of baseline error to the estimated generalization error, ratios of 2 or greater indicate that the classifier is at least twice as accurate as random guessing. Phylum level patterns were investigated using linear regression analysis of relative abundance correlations along the radial transects of polygons.

Canonical correspondence analysis (CCA) was used to identify significant environmental variables that explained the variance of the OTU-level community structure. CCA is a constrained analysis that only partitions variation that can be explained by environmental factors while using chi-square distances to perform weighted linear mapping (Oksanen et al., 2010). CCA is considered a robust and valuable analysis for ecological data because it performs well with skewed species distributions, noise, interrelated environmental variables, and violations of assumptions (Palmer, 1993). The statistical significance of explanatory variables was assessed via the adonis test, as implemented in the vegan R package (Oksanen et al., 2010). Adonis is a permutational (*n* = 999) multivariate analysis of variance test that partitions distance matrices among sources of variation. The significance of CCA model constraints were assessed by the permutation test function anova.cca. CCA tests were run when considering communities at the regional and basin scales. NMDS and CCA tests were conducted using the Vegan library (Oksanen et al., 2016) in the R programming environment.

We considered the degree and significance of spatial structuring on community-environmental relationships across regional, lake basin, and local scales via Mantel and partial Mantel tests (Mantel, 1967; Smouse et al., 1986; Legendre and Legendre, 2012). Mantel tests were conducted to assess spatial auto-correlation using Jaccard- (community composition) and Euclidean- (observed species, pH, EC, spatial) based distance matrices. Spatial distance matrices were based on geographic coordinates at the regional and basin scales and on distance from the polygon trough at local scales. Partial Mantel tests were used to compute the correlations among edaphic variables and community composition and richness while controlling for the effects of spatial structure. Mantel and partial Mantel tests were implemented using Spearman correlations within the Vegan library (Oksanen et al., 2016) and significance of results were assessed via permutational analyses (*n* = 999). Additional analysis of spatial structuring was performed using these distance matrices to create a multivariate Mantel correlogram.

We investigated the relative influence of pH versus EC on species richness using a sliding window model. To do this, we created windows (i.e., subsets) of 10 samples across each edaphic gradient and then ran the frame across the entire gradient, stepping by one sample at a time. For example, samples were ordered from lowest to highest pH and the first window contained samples 1–10 while the second window consisted of samples 2–11. The process was repeated along the EC gradient. Linear regressions of observed species in relation to pH + EC were calculated for each window and relative contributions of the edaphic variables to explainable variability (*R*^2^) were assessed using the relaimpo R package (Grömping, 2006). We used the recommended “lmg” metric, which provides a decomposition of the model explained variance into non-negative contributions while removing the effects of regression variable ordering. Results were visualized by plotting rectangles representative of the windows shaded by the proportion of variability explained along each edaphic gradient after normalization of overlapping regions. Normalization was conducted by averaging the relative contribution of each respective edaphic gradient in intervals of 5 μS/cm and 0.05 pH units.

Lastly, the effects of pH and EC as drivers of microbial community diversity and composition were assessed by Spearman rank correlations. *P*-values of environmental factors were adjusted for multiple comparisons using the Benjamini and Hochberg (1995) method.

## Results

### Sequencing Results

Pyrosequencing of 16S rRNA gene libraries resulted in 680,727 reads (6,275 ± 4,540 reads per sample, *n* = 107 samples), with a mean length of 393 ± 28 bp. Samples were rarified to 500 sequences per sample to account for uneven sequencing depth among samples. When data from all samples were considered together (regional scale), a total of 25,449 and 5,092 OTUs (97% sequence similarity) were identified in the non-rarefied and rarefied datasets, respectively. The Good’s coverage statistic of the rarified dataset ranged from 0.66 to 0.97 with an average of 0.80, indicating that the majority of diversity was detected in most samples (Good, 1953; Kemp and Aller, 2004). The rarified dataset included 5,092 OTUs that were primarily assigned to the phyla Acidobacteria (average of 28% relative abundance), Actinobacteria (9%), Bacteroidetes (19%), Deinococcus–Thermus (8%), Gemmatimonadetes (5%), and Proteobacteria (9%).

### Spatial Scale, Edaphic Gradients and Microbial Distributions

**FIGURE 1:**
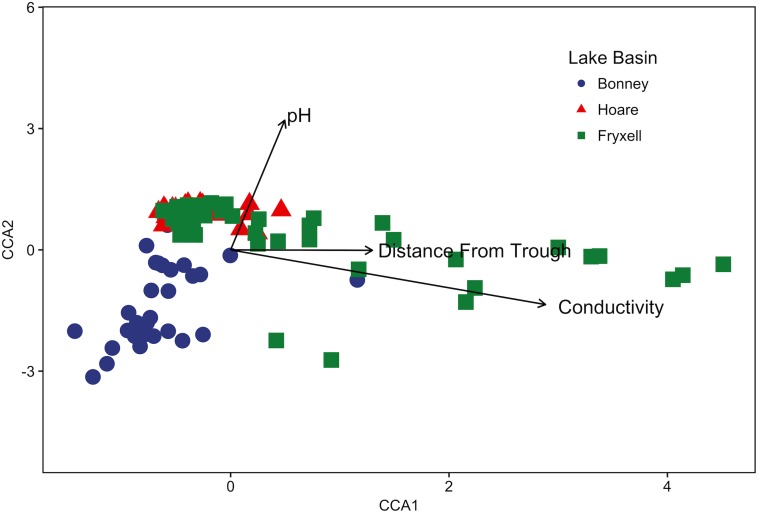
Canonical correspondence analysis (CCA) of soil samples demonstrates that community composition differs both among and within basins and that the role of conductivity, distance to trough, and pH in driving within-basin variation differs among basins. Arrows indicate direction and magnitude of significant environmental factors affecting bacterial community structure.

The relationships between edaphic gradients and the distribution of bacteria were investigated at three spatial scales; regional, basin, and local. The CCA model constructed at this geographic scale (**Table 1** and **Figure 1**) explained 17.1% of the variability of the OTU-level community matrices, leaving 82.9% of the variation unexplained (model *P* = 0.001). Additionally, adonis tests revealed that lake basin origin accounted for 25% of the community variance, EC for 14%, pH for 3%, and distance from the trough for 2% (all *P*-values ≤ 0.05). Mantel tests indicated significant spatial structuring of community and environmental variables at the regional scale (**Table 2**). Partial Mantel tests at the regional level indicated significant correlations between pH and community composition and between EC and both community composition and richness (**Table 3**). Similar results were obtained using tests based on both Jaccard and Bray–Curtis community dissimilarity matrices, so only results derived using Jaccard are reported. Additional evidence of spatial autocorrelation is evident in a Mantel correlogram (**Supplementary Figure S3**).

**Table 1:** Results of canonical correspondence analysis performed at the regional and basins scales.

Test	Statistical measure	Regional	Bonney	Hoare	Fryxell
CCA	Total Inertia	5.56	1.72	0.34	1.33
	Constrained Proportion	0.17	0.11	0.33	0.42
	Unconstrained Proportion	0.83	0.89	0.67	0.58
Anova(cca)	Model χ^2^	0.68	0.18	0.11	0.56
	Model *F*-value	9.38	1.02	5.48	8.42
	Model *P*-value	0.001^∗∗∗^	0.56	0.001^∗∗∗^	0.01^∗∗∗^
adonis	Lake Basin *R*^2^	0.25^∗∗∗^	–	–	–
	Conductivity *R*^2^	0.14^∗∗∗^	0.03	0.17^∗∗∗^	0.41^∗∗∗^
	pH *R*^2^	0.03^∗∗∗^	0.02	0.06^∗^	0.04^∗^
	Dist. from trough R^2^	0.02^∗∗^	0.03	0.14^∗∗∗^	0.04^∗^
	Residual *R*^2^	0.57	0.92	0.64	0.51

*^∗^P ≤ 0.1, ^∗∗^P ≤ 0.01, ^∗∗∗^P ≤ 0.001.*

**Table 2:** Spearman correlation coefficients of Mantel tests assess spatial structure in each edaphic property and each aspect of microbial community structure.

Scale	Location	pH	EC	Composition	Richness
Regional		0.50***	0.10**	0.44***	0.03*
Basin	Bonney	0.01	-0.01	0.28***	NS
	Hoare	0.10*	0.10*	0.13*	NS
	Fryxell	0.04	0.03	-0.01	0.14**
Local	B_1	0.83*	0.06	-0.17	0.38
	B_2	ND	ND	ND	ND
	B_3	ND	ND	ND	ND
	B_4	ND	ND	ND	ND
	B_5	0.84*	-0.03	-0.22	-0.61
	B_6	0.08	0.75**	0.27	0.02
	B_7	0.75*	-0.27	0.13	-0.23
	B_8	0.03	0.03	0.60	0.77*
	H_1	0.41	0.11	0.72*	0.42
	H_2	-0.05	0.64**	0.05	-0.06
	H_3	0.09	-0.09	0.14	0.77
	H_4	-0.17	-0.26	0.17	-0.18
	H_5	0.58*	0.86*	0.64*	-0.01
	H_6	0.20	0.71	0.83	-0.35
	H_7	0.06	-0.43	0.26	0.89*
	H_8	-0.17	-0.03	0.07	0.01
	F_1	0.12	0.92**	0.37	0.92**
	F_2	-0.20	0.99*	0.70*	0.84*
	F_3	0.22	-0.32	0.35	0.46*
	F_4	0.71*	0.03	0.02	0.11
	F_5	0.20	0.38*	-0.18	0.42*
	F_6	0.01	-0.26	0.41	-0.05
	F_7	0.22	-0.30	0.56	0.59*
	F_8	0.05	0.27	0.12	0.35

*Local scale key indicates basin_polygon number. ^∗^P ≤ 0.1, ^∗∗^P ≤ 0.01, ^∗∗∗^P ≤ 0.001, ND, not determined due to small sample size.*

**Table 3:** Partial Mantel test results, reporting Spearman correlation coefficient between each edaphic variable and aspects of community structure when controlling for geographic distance.

		Composition		Richness	
Scale	Location	pH	EC	pH	EC
Regional		0.22***	0.41***	0.01	0.23***
Basin	Bonney	0.14*	0.12	0.00	-0.08
	Hoare	0.31***	0.35***	-0.07	0.01
	Fryxell	0.13*	0.63***	0.13	0.57***
Local	B_1	0.10	0.07	0.58	0.72*
	B_2	ND	ND	ND	ND
	B_3	ND	ND	ND	ND
	B_4	ND	ND	ND	ND
	B_5	-0.42	-0.48	-0.07	-0.78
	B_6	0.20	-0.12	0.06	-0.09
	B_7	-0.36	-0.01	-0.07	-0.40
	B_8	0.66	0.66	-0.71	-0.71
	H_1	0.41*	0.76**	-0.42	0.05
	H_2	0.46	0.46	-0.50	-0.10
	H_3	-0.22	-0.25	0.57	0.33
	H_4	0.26	0.88**	-0.24	0.00
	H_5	0.36	0.02	0.27	-0.05
	H_6	-0.15	-0.13	0.90*	-0.50
	H_7	0.51	0.43	-0.04	0.22
	H_8	0.09	0.08	-0.50	-0.13
	F_1	-0.29	0.62*	0.20	0.92**
	F_2	0.24	0.71*	-0.70	-0.62
	F_3	0.17	-0.36	0.55	-0.29
	F_4	-0.46	0.56*	-0.62	0.69*
	F_5	-0.20	0.93**	-0.29	0.96*
	F_6	0.52*	0.64*	0.26	0.61
	F_7	-0.13	0.02	-0.19	-0.39
	F_8	-0.47	0.71**	-0.55	0.76*

*Local scale key indicates basin_polygon number. ^∗^P ≤ 0.1, ^∗∗^P ≤ 0.01, ^∗∗∗^P ≤ 0.001, ND, not determined due to small sample size.*

At the basin scale, soil pH and EC varied significantly (Kruskal–Wallis; pH: *P* < 0.001, EC: *P* < 0.001) (**Table 4**). The pH values were least alkaline in the Bonney Basin (mean 8.77, ±SE 0.06), intermediate in the Fryxell Basin (mean 9.57, ±SE 0.08), and most basic in the Hoare Basin (10.03, ±SE 0.04) (**Table 4**). In contrast, soil EC values were lowest in Hoare Basin (144 μS/cm, ± SE 10), intermediate in Bonney Basin (361 μS/cm, ±SE 54), and highest in Fryxell Basin (788 μS/cm, ±SE 135) (**Table 4**). Alpha diversity also varied significantly among basins (Kruskal–Wallis; observed species: *P* < 0.05 and inverse Simpson: *P* < 0.001) (**Table 4**). The highest average microbial richness, as measured by the number of observed species (OTUs), was found in the Lake Hoare basin (166 ± 6) and the lowest in the Lake Fryxell basin (136 ± 7). The inverse Simpson diversity index, which incorporates species evenness, was highest (43.31 ± 4.55) in the Lake Bonney basin soils and lowest (20.68 ± 2.16) in the Lake Fryxell Basin soils. Both indices indicated that the Fryxell basin communities were the least diverse.

**Table 4:** Mean ± standard error of soil geochemical properties and alpha diversity values for soils collected from each lake basin.

Parameter	Lake basin	Minimum	Maximum	Range	Mean	*SE*
pH^∗^	Bonney	7.64	9.33	1.69	8.77^a^	0.06
	Hoare	9.39	10.40	1.01	10.03^c^	0.04
	Fryxell	8.40	10.35	1.95	9.57^b^	0.08
						
Conductivity^∗^	Bonney	119	1636	1517	361^a^	54
	Hoare	56	296	40	144^a^	10
	Fryxell	86	2808	2722	788^b^	135
						
Observed species^∗^	Bonney				146^ab^	9
	Hoare				166^b^	6
	Fryxell				136^a^	7
						
Inverse Simpson^∗^	Bonney				43.3^c^	4.6
	Hoare				30.9^b^	2.3
	Fryxell				20.7^a^	2.2

*Ranges of edaphic factors (minimum–maximum, difference) are reported inside parentheses. ^∗^Indicates significant difference in raw data among basins (P < 0.05). Basin means with same superscript letter are not statistically different after adjusting for multiple comparisons.*

**FIGURE 2:**
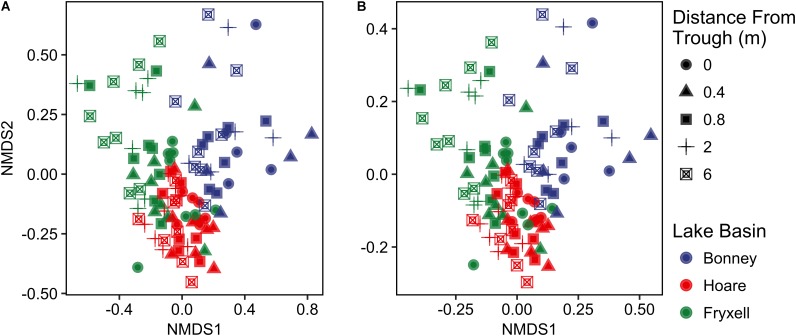
Non-metric multidimensional scaling plots created using **(A)** Bray–Curtis and **(B)** Jaccard distances show distinctiveness of communities among basins.

Differences were observed in the overall taxonomic composition of the soils from the various basins. Soils from Lake Bonney Basin had the most even distribution of phyla, containing 4-15% relative abundances of Acidobacteria, Actinobacteria, Bacteroidetes, Firmicutes, Planctomycetes, Proteobacteria, Verrucomicrobia, and Deinococcus–Thermus (**Supplementary Figure S2**). Lake Fryxell basin soils were dominated by Acidobacteria (28%), Bacteroidetes (24%), and Deinococcus–Thermus (15%). Acidobacteria were especially dominant in the Lake Hoare basin soils (43%), which also included Bacteroidetes (18%), Actinobacteria (9%), and Verrucomicrobia (7%). Bacterial community composition varied significantly among lake basins as evidenced by statistically significant clustering by basin (ANOSIM R statistic = 0.47, *P* = 0.001) using both Bray–Curtis (Legendre and Gallagher, 2001) and Jaccard distances (**Figure 2**), in addition to high random forests classification ratios (ratio of 14.1). Basin-specific CCA models were only significant for communities within the Lake Hoare and Fryxell basins (**Table 1**). EC explained the most variation in the Lake Fryxell basin (adonis: *R*^2^ = 0.41), while pH and distance from the trough only explained 4% of variation. Within the Hoare basin, EC explained 17% of variation, distance from the trough explained 14%, and pH accounted for 6%. Partial Mantel tests at the regional level indicated significant correlations between pH and community composition in all three basins, EC and composition in Hoare and Fryxell basins, and EC and richness in Fryxell Basin (**Table 3**).

**FIGURE 3:**
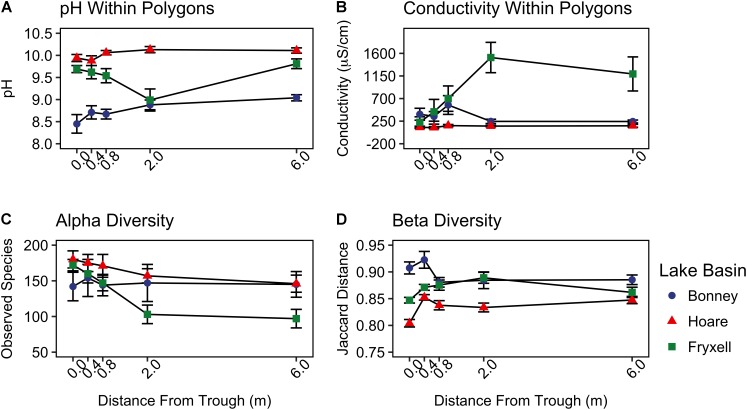
Basin scale spatial variability of **(A)** pH, **(B)** conductivity, **(C)** alpha diversity (observed species), and **(D)** beta diversity (Jaccard distance) (mean ± standard error) along radial transects of polygons. Beta diversity was calculated by pooling samples from each distance from the trough within each lake basin.

**FIGURE 4:**
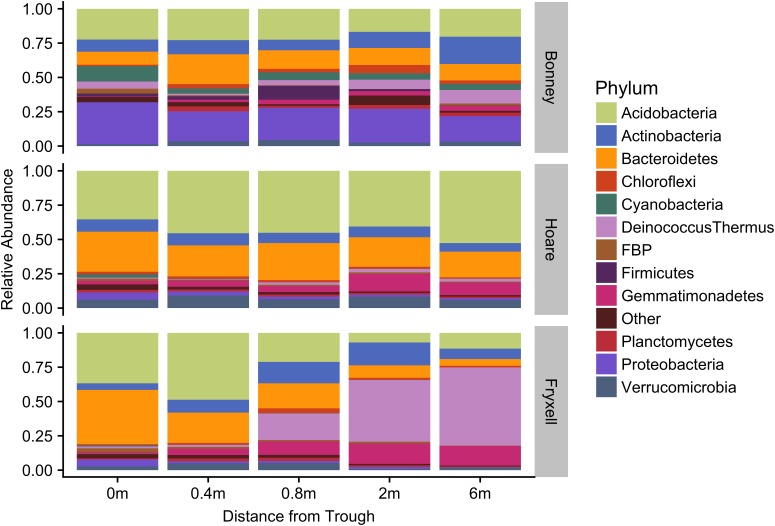
Median relative abundance of top phyla within basins along polygon radial transects (filtered to minimum 1% average relative abundance).

At the local scale, soil pH varied significantly along the polygon transects only in the Lake Bonney basin (Kruskal–Wallis; *P* = 0.0358) (**Figure 3** and **Supplementary Table S1**), increasing from an average of 8.45 to 9.04 from the trough to the polygon center. Soil EC only varied significantly along transects in the Lake Fryxell basin (Kruskal–Wallis; *P* = 0.036), increasing from 228 μS/cm at the trough to 1,190 μS/cm in the center. Along the polygon transects, the highest number of observed species was found within the Lake Hoare polygon troughs while the lowest was in the center of Lake Fryxell polygons. Lake Bonney basin transects generally had the highest inverse Simpson values and Lake Fryxell basin transects had the lowest. Both metrics decreased toward the center of polygons within the Lake Fryxell basin (Kruskal–Wallis; observed species: *P* = 0.0006, inverse Simpson: *P* = 0.0028). The relative composition of phyla along the polygon transects within Bonney and Hoare basins were stable and indistinguishable (random forests ratios of 1.0 and 1.7, respectively, **Figure 4**). In contrast, communities along the transects in the Fryxell basin soils diverged significantly (random forests ratio of 2.1) as Deinococcus–Thermus and Gemmatimonadetes increased in relative abundance toward the center of the polygons while Acidobacteria and Bacteroidetes decreased. Populations of Acidobacteria and Actinobacteria were inversely correlated, as were Bacteroidetes versus Deinococcus–Thermus and Gemmatimonadetes (**Table 5**). Both Bacteroidetes and Proteobacteria, as well as Gemmatimonadetes and Deinococcus–Thermus were positively correlated. Correlations between soil chemistry and community structure at the local level were only significant for some transects in some basins. In the Fryxell basin, significant correlations were observed between community composition and EC along six out of eight transects, and between richness and EC at four out of eight transects (**Table 3**). Raw edaphic and alpha diversity values are reported in **Supplementary Table S2**.

**Table 5:** Statistical metrics of significant linear regressions comparing phyla relative abundances along polygon transects within the Fryxell basin.

Independent variable	Dependent variable	*X* – Intercept	Correlation coefficient	*R*^2^
Acidobacteria	Actinobacteria	0.43^∗∗^	-1.12^∗^	0.74^∗^
Bacteroidetes	Gemmatimonadetes	0.31^∗∗∗^	-2.12^∗∗^	0.93^∗∗^
Bacteroidetes	Proteobacteria	0.01	0.25^∗^	0.73^∗^
Bacteroidetes	Deinococcus–Thermus	0.26^∗∗∗^	-0.70^∗^	0.82^∗^
Gemmatimonadetes	Deinococcus–Thermus	0.03^∗^	0.31^∗^	0.82^∗^

*^∗^P ≤ 0.1, ^∗∗^P ≤ 0.01, ^∗∗∗^P ≤ 0.001.*

### Threshold Effects and Phylum-Specific Patterns

**FIGURE 5:**
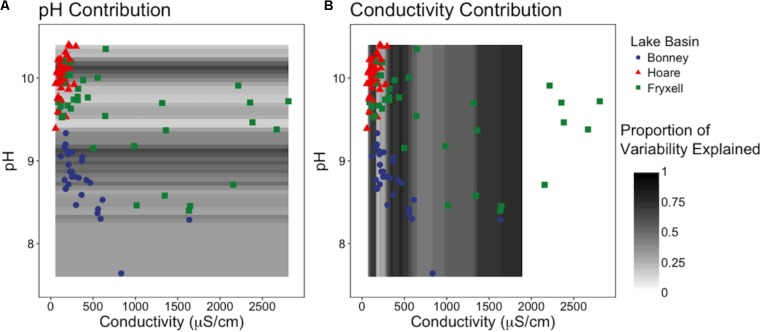
Sliding window model: Relative contributions of pH and conductivity to explainable variation in observed species across **(A)** the pH gradient and **(B)** the conductivity gradient. Models reveals that EC is generally more influential to community diversity and composition, but when EC is low, pH is more important.

Our sliding window model constructed at the regional scale simultaneously assessed the effects of pH and EC on richness and indicated that EC generally explained the majority of variation in the number of observed species (**Figure 5**). EC was the more influential edaphic variable across intervals from 80–165, 290–640, and at 1000 μS and above (**Figure 5B**). In contrast, pH explained more variation in small windows from 8.95–9.1 to 10.05–10.15 (**Figure 5A**).

**FIGURE 6:**
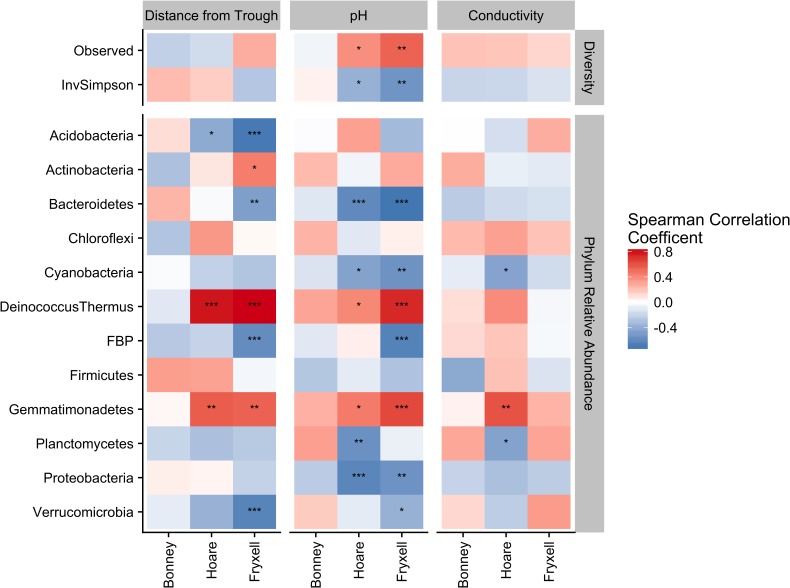
Assessing relative influence of edaphic factors over alpha diversity **(top)** and major phyla relative abundances **(bottom)** using Spearman rank correlations, revealing that phyla tend to respond in a similar direction regardless of basin. ^∗^*P* ≤ 0.05, ^∗∗^*P* ≤ 0.01, ^∗∗∗^*P* ≤ 0.001. *P*-values of distance from trough, pH, and conductivity adjusted for multiple comparison using Benjamini and Hochberg (1995).

Phylum-specific spearman rank correlations were calculated to determine the proportion of variance in alpha diversity and phyla relative abundance that could be explained by distance from the polygon trough, pH, and EC (**Figure 6**). Significant correlations between distance from trough and alpha diversity were found within the Lake Hoare and Fryxell basin soils based on positive correlations between distance and observed species and negative correlations between distance and inverse Simpson (all *P* < 0.05). Significant correlations to phyla relative abundance were also only found in the Hoare and Fryxell lake basins. In all cases where a phylum was significantly correlated to an edaphic gradient in more than one basin, the direction of the correlation was consistent between basins; for example, Deinococcus–Thermus was positively correlated with EC in both Hoare and Fryxell basins (**Figure 6**).

## Discussion

To better understand the relationship between bacterial species distribution patterns and the environmental factors that shape them, we performed a spatially stratified examination of the inherent edaphic gradients within the cold, dry, and oligotrophic polar desert ecosystem of the MDV, Antarctica. This study focused on the relationships among edaphic pH and EC gradients on bacterial communities as they vary across local, lake basin, and regional scales. The simplified trophic structure and high spatial physiochemical heterogeneity of MDV soils provides an ideal model system in which to study natural, low-complexity microbial community-environment interactions and thus better understand the underlying scale-dependent processes that structure these communities. By minimizing the effects of biotic interactions, we can more directly address the poorly understood impacts of environmental heterogeneity and spatial scale on microbial community composition and diversity. Our findings corroborate previous studies of soil bacterial communities within the lake basins of Taylor Valley by describing high spatial variability and linking soil microbial community structure to edaphic geochemical gradients (Barrett et al., 2006; Niederberger et al., 2008; Smith et al., 2010; Zeglin et al., 2011; Lee et al., 2012; Sokol et al., 2013; Van Horn et al., 2013). However, this is the first study to use the inherent edaphic gradients within soil polygons to investigate the effects of spatial scale, environmental heterogeneity, and landscape context on bacterial community structure.

### Environmental Gradients at Different Scales

Edaphic gradients, which strongly affect the spatial patterning of soil microbial communities (Barrett et al., 2006; Fierer and Jackson, 2006; Lozupone and Knight, 2007; Niederberger et al., 2008, 2015; Smith et al., 2010; Zeglin et al., 2011; Lee et al., 2012; Sokol et al., 2013; Van Horn et al., 2013), occur across different spatial scales due to variation in the underlying drivers producing these gradients. For example, broad-scale edaphic gradients can be caused by differences in topography, climate, and geologic history that occur across landscapes (Jenny, 1941; Sinsabaugh et al., 2008; Townsend et al., 2008). The variations in edaphic conditions that we observed between lake basins in the MDV were most clearly illustrated by the absence of overlap between samples from different lake basins in our pH versus EC bi-plots (**Figure 5**). These observations are an example of landscape-scale gradients likely due to differences in parent geology, glacial till sequence, and paleo-lacustrine organic matter deposition (Péwé, 1960; Burkins et al., 2000).

While broad-scale gradients create a general template for the formation of biological communities, fine-scale factors such as soil structure, microclimates, topography, and transition zones between habitats (ex. riparian zones), can superimpose local-scale gradients on top of regional patterns (Ettema and Wardle, 2002). In the MDV, fine-scale gradients in edaphic factors including soil moisture, organic matter, pH, EC, ions, and nutrients have been observed near snowpacks (Gooseff et al., 2003; Van Horn et al., 2013), lake margins (Northcott et al., 2009; Zeglin et al., 2009), hyporheic zones (Barrett et al., 2009; Northcott et al., 2009; Zeglin et al., 2009; Niederberger et al., 2015), ponds (Moorhead et al., 2003), and mummified seals (Tiao et al., 2012). In this study, the effects of the physical processes inherent to polygon formation, (e.g., frost-sorting and aeolian deposition, Kessler et al., 2001; Bockheim, 2002; Kessler and Werner, 2003; Barrett et al., 2004) created local-scale gradients within some (Fryxell), but not all (Bonney and Hoare) basins. Thus, both the basin- and local-scale gradients for pH and EC provided an opportunity to study the effects of edaphic gradients on microbial community diversity and composition.

### Bacterial Community Responses to Gradients

In this study, we focused on bacterial community responses to pH and EC gradients because these edaphic parameters are master drivers of microbial community structure and diversity (Fierer and Jackson, 2006; Lozupone and Knight, 2007). High soil salinity increases water limitation by controlling total ion concentrations, and therefore the total water potential of the soil. Salinity can exert additional biological stress on soil microbes by osmotically increasing intracellular ion concentrations to potentially toxic levels resulting in decreases in respiration, critical enzyme activity, and nitrogen and carbon cycling (Frankenberger and Bingham, 1982; Zahran, 1997). The exact mechanism by which pH exerts its effects on microbes is less well defined, though two possible explanations have been proposed. First, pH may be an amalgam of other edaphic characteristics such as nutrient concentrations, cationic metal solubility, organic matter content, moisture, and salinity, and it is these variables that directly impact the soil communities (Lauber et al., 2009). Alternatively, severe pH gradients may create a selective advantage for species with increased tolerance to pH extremes (Lauber et al., 2009).

As described above, our sampling encompassed both broad- and local-scale variation in these primary edaphic variables, along with the subsequent impacts to bacterial community structure. Similar to other studies (e.g., Fierer and Jackson, 2006; Lauber et al., 2009), broad-scale environmental differences created distinct communities in spatially distant, but cohesive, areas (lake basins) based on ordination analyses (**Figures 2, 5**). In particular, the Lake Bonney communities were differentiated from those in the other two lake basins, and the Lake Bonney soils had the lowest minimum, maximum, and mean soil pH. This result, coupled with the overall low within- and high between-basin variation in pH, suggests that this variable is particularly important in shaping the baseline community found in different regions of the MDV. Superimposed upon the broad-scale pH pattern, local polygon-scale conductivity gradients appear to drive local community composition, with predictable increases in Deinococcus–Thermus and Gemmatimonadetes and decreases in Acidobacteria, Bacteroidetes, and Proteobacteria with increasing EC (**Table 5** and **Figures 3, 4**). These findings were particularly strong in Lake Fryxell basin, indicating that edaphic variations may elicit sufficient physiological stress to result in community changes due to environmental filtering and competitive interactions, as observed elsewhere (Weiher and Keddy, 2001).

Our findings of both local- and broad-scale edaphic and community patterns, contrast with several previous studies both within and outside of the MDV that found diverse or non-existent patterning at local scales and coherent patterns at larger scales (Barrett et al., 2004; O’Brien et al., 2016). We propose that a key to understanding this discrepancy is the degree of edaphic heterogeneity captured within our study. More specifically, not only is the presence of gradients important in structuring microbial communities, but the length or severity of the gradient is also crucial. For example, Lake Fryxell basin soils have the highest average EC (788 ± 135 μS) and range (86–2808 μS) (**Table 4**) and the bacterial communities at Lake Fryxell were the most highly responsive to EC (**Figures 1, 5**). Furthermore, the strength of relationships among edaphic conditions and microbial communities depended on the magnitude of the heterogeneity exhibited at each scale that was analyzed (**Table 1**). Although the magnitude of relationships among community structure and edaphic factors varied among basins, the direction of change remained constant (**Figure 6**). This implies that, regardless of location, phyla are responding similarly to edaphic conditions. However, because the core communities were different due to the broad-scale underlying factors, communities from different basins did not appear to converge even at the extreme end of the edaphic ranges.

Our results suggest that Deinococcus–Thermus was likely the only phylum thriving in the high EC soils of the Lake Fryxell polygon centers. These organisms have previously been associated with low productivity soils (Niederberger et al., 2008). Deinococcus–Thermus had the largest variation across all scales, second only to Proteobacteria (**Supplementary Figure S2**). Interestingly, Proteobacteria have been identified as constituting a substantial proportion of the active communities in the MDV by studies using stable isotope probing (Schwartz et al., 2014) and RNA sequencing (Buelow et al., 2016). Thus, we can surmise that Proteobacteria and Deinococcus–Thermus are active and adapting to environmental conditions within our study system. Furthermore, the two aforementioned activity-based studies indicated that Acidobacteria and Bacteroidetes were largely inactive. Our study found comparably low variation for these phyla across spatial scales, indicating that perhaps they (or their relic DNA) had a more cosmopolitan distribution that decreased in relative abundance when environmental conditions better suited more specialized taxa, i.e., high EC selection of Deinococcus–Thermus.

Together, these results suggest several consistent patterns with respect to interactions between environmental gradients and bacterial community structure. First, sufficiently long gradients allowed a greater number of niches (and therefore a greater number of taxa), resulting in variation in community composition along the gradients. These niches may be due to differences physiological stresses or other factors correlated to the abiotic gradient (Okie et al., 2015). We found that at any scale, there was potential for thresholds of effect: we observed local-scale thresholds within the Fryxell lake basin soils, but basin-scale thresholds in pH between Lake Bonney and Lake Hoare soils. Additionally, these edaphic factors did not work in isolation, but instead interacted synergistically. Further investigation into the relative contributions of EC and pH to microbial richness at the regional-scale revealed the dominance of EC (**Figure 5B**), while pH explained more variation in small windows that correspond to EC levels below 500 μS (**Figure 5A**). This not only highlights the importance of gradient severity, but reveals that when EC is low, pH is more influential to community diversity and composition. Thus, it appears that environmental gradient length organizes soil bacterial communities. This conclusion is especially interesting considering the discrepancies over the suggested dominant environmental drivers reported in the literature. For example, Fierer and Jackson (2006) suggest that soil bacterial diversity is primarily correlated to pH, whereas Lozupone and Knight (2007) found diversity strongly correlates to soil salinity, but not pH. Our study encompassed almost neutral to very basic soils, ranging from ∼7.6 to 10.4 while Fierer and Jackson (2006) studied a pH range of 3.5 to 9, and did not consider soil salinity. We note that it is plausible that microbial communities are more responsive to acidity than alkalinity due to the complexities involved in the physiological adaptations toward acidophily (Colman et al., 2017). The pH and salinity ranges in the Lozupone and Knight (2007) study are not reported, and samples were binned into “saline” and “non-saline” groups. Thus, it is possible there are no actual contradictions in the conclusions given by these research groups but that these reportedly differing results were due to limitations of the edaphic gradients present in the different studies.

## Conclusion

These findings stress the importance of a spatially explicit experimental design and recognition of the inherent gradients across a variety of spatial scales. In particular, effort is needed to sample the entirety of gradients present at each relevant spatial scale before reaching conclusions about the distribution of organisms or relationships among communities and environmental factors. Failure to do so may lead to spurious inferences or results that are artifacts of limited and unrepresentative data. Random sampling with the objective of capturing an unbiased representation of soil heterogeneity may capture edaphic ranges but will not capture the local structure needed to inform our understanding of ecological processes. For instance, we captured clear patterns at scales less than 6 m in Lake Fryxell soils because we had sufficient EC gradients to elicit physiological response. The substantial local EC gradients resulted from the physical processes of polygon formation and significantly affected larger-scale patterning. While environmental extremes may be less frequent, they play an important role in structuring biota across the landscape, even in the physiologically challenging environment posed by the MDV. Furthermore, we observed coherent local-scale patterns because the bacterial communities were (1) diverse, (2) active, and (3) adapted to the local environment. Barrett et al. (2004) did not observe clear local-scale patterns, potentially because the edaphic gradients sampled did not impart sufficient physiological stress on the nematodes, or perhaps because the eukaryotic community was not diverse enough and many samples did not contain organisms. In contrast, the ubiquity of bacteria in these soils allows us to see an additional aspect of environment-community interactions, that is, the effects of environmental filtering across finer spatial scales.

We conclude that a combination of local-scale polygon mechanisms as well as regional-scale geological histories drove changes in edaphic gradients that played a large role in determining the microbial community composition and diversity within the McMurdo Dry Valleys of Antarctica. Our results suggest that the relative importance of pH versus EC in structuring microbial communities is contextually related to the length and severity of edaphic gradients and the spatial scale of sampling, creating a framework in which to interpret conflicting literature.

## Author Contributions

Funding was secured by CT-V, DVH, and ES. DVH conceived and designed the experiments. DVH, DC, TM, and HB performed the fieldwork. KF processed the samples for DNA sequencing. DVH, CT-V, and KF analyzed the data. KF, DVH, and CT-V wrote the manuscript with input from all the authors.

## Conflict of Interest Statement

The authors declare that the research was conducted in the absence of any commercial or financial relationships that could be construed as a potential conflict of interest.
