# Supplementary material for: Local and Regional Scale Heterogeneity Drive Bacterial Community Diversity and Composition in a Polar Desert
Source: Front Microbiol. 2018 Aug 21;9:1928. doi: 10.3389/fmicb.2018.01928 (PMC6110917; doi:10.3389/fmicb.2018.01928)
Supplement: Supplementary file 1 [file Table_1.DOCX]

Supplemental Table 1: Mean ± standard error of soil geochemical properties and alpha diversity values of soils collected from each distance from the polygon trough within each lake basin. Means with the same superscript letter are not statistically different after adjusting for multiple comparisons.

| Lake Basin | Distance from Trough (m) | pH | | | Conductivity (μS) | | | Observed Species | | | Inv. Simpson | | |
| --- | --- | --- | --- | --- | --- | --- | --- | --- | --- | --- | --- | --- | --- |
| Bonney | 0 | 8.45^a^ | ± | 0.21 | 388 | ± | 119 | 142 | ± | 20 | 41.6 | ± | 11.3 |
|  | 0.4 | 8.71^ab^ | ± | 0.15 | 345 | ± | 96 | 154 | ± | 26 | 39.7 | ± | 13.1 |
|  | 0.8 | 8.67^ab^ | ± | 0.11 | 571 | ± | 187 | 144 | ± | 15 | 56.5 | ± | 9.6 |
|  | 2 | 8.88^ab^ | ± | 0.11 | 246 | ± | 43 | 147 | ± | 26 | 35.4 | ± | 12.3 |
|  | 6 | 9.04^b^ | ± | 0.07 | 243 | ± | 29 | 145 | ± | 18 | 40.0 | ± | 7.9 |
|  |  |  |  |  |  |  |  |  |  |  |  |  |  |
| Hoare | 0 | 9.94 | ± | 0.08 | 127 | ± | 23 | 180 | ± | 12 | 40.9 | ± | 6.0 |
|  | 0.4 | 9.88 | ± | 0.11 | 121 | ± | 20 | 175 | ± | 12 | 31.8 | ± | 4.5 |
|  | 0.8 | 10.06 | ± | 0.05 | 162 | ± | 22 | 171 | ± | 16 | 35.9 | ± | 5.1 |
|  | 2 | 10.13 | ± | 0.07 | 152 | ± | 15 | 157 | ± | 10 | 22.4 | ± | 4.5 |
|  | 6 | 10.11 | ± | 0.06 | 158 | ± | 29 | 146 | ± | 12 | 25.8 | ± | 3.3 |
|  |  |  |  |  |  |  |  |  |  |  |  |  |  |
| Fryxell | 0 | 9.69 | ± | 0.08 | 228^a^ | ± | 108 | 172^c^ | ± | 8 | 30.7^b^ | ± | 3.7 |
|  | 0.4 | 9.62 | ± | 0.15 | 437^ab^ | ± | 246 | 160^c^ | ± | 13 | 25.6^b^ | ± | 4.8 |
|  | 0.8 | 9.54 | ± | 0.16 | 698^ab^ | ± | 253 | 147^bc^ | ± | 11 | 23.8^bc^ | ± | 5.0 |
|  | 2 | 8.99 | ± | 0.25 | 1517^b^ | ± | 302 | 103^ab^ | ± | 13 | 13.9^bc^ | ± | 2.8 |
|  | 6 | 9.81 | ± | 0.11 | 1190^b^ | ± | 339 | 97^a^ | ± | 13 | 9.2^ac^ | ± | 3.0 |
