# Supplementary material for: Local and Regional Scale Heterogeneity Drive Bacterial Community Diversity and Composition in a Polar Desert
Source: Front Microbiol. 2018 Aug 21;9:1928. doi: 10.3389/fmicb.2018.01928 (PMC6110917; doi:10.3389/fmicb.2018.01928)
Supplement: Supplementary file 2 [file Table_2.DOCX]

Supplemental Table 2: Raw soil geochemical results and alpha diversity values of soils collected from each distance from the polygon trough within each lake basin. Data are excluded from samples with less than 500 sequencing reads.

| Lake Basin | Polygon Number | Distance from Trough (m) | pH | Conductivity (μS) | Observed Species | Inv. Simpson |
| --- | --- | --- | --- | --- | --- | --- |
| Bonney | 1 | 0 | 8.47 | 299 | 147 | 20.1 |
|  |  | 0.4 | 8.42 | 558 | 170 | 69.8 |
|  |  | 0.8 | 8.37 | 552 | 148 | 27.3 |
|  |  | 2 | 9.17 | 163 | 60 | 6.4 |
|  |  | 6 | 9.33 | 178 | 90 | 18.8 |
|  |  |  |  |  |  |  |
|  | 2 | 0 |  |  |  |  |
|  |  | 0.4 | 9.06 | 119 | 174 | 16.2 |
|  |  | 0.8 | 8.53 | 612 | 143 | 61.7 |
|  |  | 2 |  |  |  |  |
|  |  | 6 | 8.87 | 248 | 125 | 42.7 |
|  |  |  |  |  |  |  |
|  | 3 | 0 |  |  |  |  |
|  |  | 0.4 |  |  |  |  |
|  |  | 0.8 | 8.74 | 466 | 115 | 47.2 |
|  |  | 2 | 8.79 | 331 | 134 | 15.3 |
|  |  | 6 | 8.66 | 176 | 131 | 58.0 |
|  |  |  |  |  |  |  |
|  | 4 | 0 |  |  |  |  |
|  |  | 0.4 |  |  |  |  |
|  |  | 0.8 |  |  |  |  |
|  |  | 2 |  |  |  |  |
|  |  | 6 | 9.04 | 371 | 112 | 16.8 |
|  |  |  |  |  |  |  |
|  | 5 | 0 | 7.64 | 832 | 172 | 73.2 |
|  |  | 0.4 | 8.30 | 589 | 51 | 1.3 |
|  |  | 0.8 | 8.29 | 1636 | 114 | 37.4 |
|  |  | 2 |  |  |  |  |
|  |  | 6 | 9.10 | 255 | 164 | 68.6 |
|  |  |  |  |  |  |  |
|  | 6 | 0 | 8.72 | 209 | 98 | 25.1 |
|  |  | 0.4 | 8.95 | 210 | 202 | 54.8 |
|  |  | 0.8 | 8.87 | 226 | 214 | 101.4 |
|  |  | 2 | 8.59 | 364 | 177 | 76.9 |
|  |  | 6 | 8.90 | 367 | 212 | 38.3 |
|  |  |  |  |  |  |  |
|  | 7 | 0 | 8.67 | 177 | 98 | 24.7 |
|  |  | 0.4 | 8.81 | 249 | 174 | 56.3 |
|  |  | 0.8 | 8.81 | 287 | 134 | 44.9 |
|  |  | 2 | 8.77 | 163 | 214 | 38.2 |
|  |  | 6 | 9.20 | 175 | 160 | 64.6 |
|  |  |  |  |  |  |  |
|  | 8 | 0 | 8.77 | 421 | 196 | 65.1 |
|  |  | 0.4 |  |  |  |  |
|  |  | 0.8 | 9.12 | 199 | 232 | 75.6 |
|  |  | 2 | 9.08 | 206 | 150 | 40.4 |
|  |  | 6 | 9.12 | 196 | 80 | 12.5 |
|  |  |  |  |  |  |  |
| Hoare | 1 | 0 | 10.12 | 221 | 219 | 54.5 |
|  |  | 0.4 | 9.73 | 123 | 195 | 32.9 |
|  |  | 0.8 | 10.00 | 122 | 203 | 41.0 |
|  |  | 2 | 10.04 | 110 | 137 | 7.6 |
|  |  | 6 | 9.53 | 165 | 169 | 31.9 |
|  |  |  |  |  |  |  |
|  | 2 | 0 | 9.93 | 186 | 195 | 57.1 |
|  |  | 0.4 | 9.53 | 177 | 202 | 36.2 |
|  |  | 0.8 | 9.99 | 166 | 221 | 46.0 |
|  |  | 2 | 9.88 | 122 | 207 | 40.5 |
|  |  | 6 | 10.17 | 116 | 173 | 24.8 |
|  |  |  |  |  |  |  |
|  | 3 | 0 |  |  |  |  |
|  |  | 0.4 | 10.10 | 141 | 181 | 14.5 |
|  |  | 0.8 | 10.11 | 159 | 177 | 28.1 |
|  |  | 2 | 10.31 | 175 | 189 | 41.0 |
|  |  | 6 | 10.06 | 136 | 180 | 25.1 |
|  |  |  |  |  |  |  |
|  | 4 | 0 | 10.13 | 145 | 208 | 46.0 |
|  |  | 0.4 | 10.22 | 223 | 194 | 43.7 |
|  |  | 0.8 | 10.27 | 188 | 203 | 43.9 |
|  |  | 2 | 10.40 | 211 | 177 | 32.6 |
|  |  | 6 | 10.18 | 184 | 196 | 39.5 |
|  |  |  |  |  |  |  |
|  | 5 | 0 | 10.14 | 114 | 198 | 49.3 |
|  |  | 0.4 | 10.22 | 87 | 144 | 15.9 |
|  |  | 0.8 | 10.23 | 114 | 122 | 14.3 |
|  |  | 2 | 10.38 | 226 | 139 | 20.4 |
|  |  | 6 | 10.38 | 296 | 130 | 14.2 |
|  |  |  |  |  |  |  |
|  | 6 | 0 | 9.75 | 82 | 144 | 39.9 |
|  |  | 0.4 | 10.08 | 76 | 212 | 51.1 |
|  |  | 0.8 |  |  |  |  |
|  |  | 2 | 10.06 | 74 | 116 | 18.3 |
|  |  | 6 | 10.18 | 117 | 147 | 25.5 |
|  |  |  |  |  |  |  |
|  | 7 | 0 | 9.59 | 84 | 154 | 23.0 |
|  |  | 0.4 | 9.39 | 57 | 161 | 33.2 |
|  |  | 0.8 | 9.93 | 108 | 155 | 52.6 |
|  |  | 2 | 9.97 | 107 | 108 | 8.6 |
|  |  | 6 | 9.97 | 95 | 141 | 17.9 |
|  |  |  |  |  |  |  |
|  | 8 | 0 | 9.93 | 56 | 142 | 15.7 |
|  |  | 0.4 | 9.77 | 87 | 114 | 26.7 |
|  |  | 0.8 | 9.92 | 275 | 113 | 25.3 |
|  |  | 2 | 9.95 | 125 | 132 | 25.9 |
|  |  | 6 | 9.86 | 210 | 155 | 33.8 |
|  |  |  |  |  |  |  |
| Fryxell | 1 | 0 | 9.71 | 86 | 208 | 50.2 |
|  |  | 0.4 | 9.54 | 149 | 192 | 20.9 |
|  |  | 0.8 | 9.73 | 319 | 181 | 47.5 |
|  |  | 2 | 8.40 | 1632 | 87 | 10.1 |
|  |  | 6 | 9.46 | 2383 | 45 | 2.6 |
|  |  |  |  |  |  |  |
|  | 2 | 0 | 9.69 | 120 | 192 | 39.6 |
|  |  | 0.4 | 9.74 | 271 | 197 | 55.8 |
|  |  | 0.8 | 9.77 | 317 | 167 | 34.3 |
|  |  | 2 |  |  |  |  |
|  |  | 6 | 9.7 | 2355 | 60 | 9.1 |
|  |  |  |  |  |  |  |
|  | 3 | 0 | 9.95 | 152 | 155 | 26.1 |
|  |  | 0.4 | 10.03 | 222 | 186 | 26.2 |
|  |  | 0.8 | 9.87 | 323 | 170 | 30.9 |
|  |  | 2 | 10.20 | 165 | 114 | 6.63 |
|  |  | 6 | 10.19 | 240 | 103 | 3.7 |
|  |  |  |  |  |  |  |
|  | 4 | 0 | 9.75 | 92 | 190 | 33.0 |
|  |  | 0.4 | 9.63 | 236 | 178 | 22.9 |
|  |  | 0.8 | 9.76 | 437 | 153 | 22.0 |
|  |  | 2 | 9.54 | 643 | 144 | 16.1 |
|  |  | 6 | 9.97 | 383 | 145 | 10.8 |
|  |  |  |  |  |  |  |
|  | 5 | 0 | 9.72 | 115 | 153 | 19.7 |
|  |  | 0.4 | 9.59 | 93 | 139 | 18.8 |
|  |  | 0.8 | 9.91 | 2215 | 86 | 5.0 |
|  |  | 2 | 9.37 | 1360 | 111 | 22.0 |
|  |  | 6 | 9.38 | 2669 | 64 | 2.8 |
|  |  |  |  |  |  |  |
|  | 6 | 0 | 9.94 | 160 | 171 | 32.1 |
|  |  | 0.4 | 8.71 | 2156 | 91 | 29.0 |
|  |  | 0.8 | 8.58 | 1346 | 115 | 9.6 |
|  |  | 2 | 8.45 | 1646 | 97 | 18.7 |
|  |  | 6 | 9.70 | 1316 | 80 | 5.5 |
|  |  |  |  |  |  |  |
|  | 7 | 0 | 9.18 | 984 | 163 | 21.5 |
|  |  | 0.4 | 9.67 | 200 | 129 | 9.3 |
|  |  | 0.8 | 9.53 | 120 | 148 | 12.9 |
|  |  | 2 | 8.46 | 1014 | 124 | 14.0 |
|  |  | 6 | 10.00 | 553 | 99 | 11.2 |
|  |  |  |  |  |  |  |
|  | 8 | 0 | 9.66 | 115 | 141 | 23.3 |
|  |  | 0.4 | 10.04 | 169 | 166 | 21.9 |
|  |  | 0.8 | 9.15 | 499 | 154 | 28.2 |
|  |  | 2 | 9.72 | 2808 | 55 | 2.4 |
|  |  | 6 | 10.35 | 649 | 106 | 4.9 |
