# Supplementary material for: Local and Regional Scale Heterogeneity Drive Bacterial Community Diversity and Composition in a Polar Desert
Source: Front Microbiol. 2018 Aug 21;9:1928. doi: 10.3389/fmicb.2018.01928 (PMC6110917; doi:10.3389/fmicb.2018.01928)
Supplement: Supplementary file 3 [file Data_Sheet_1.docx]

Supplemental Figure 1: Map of sampling locations. Red stars indicate approximate locations of polygons within the Lake Bonney (LB), Lake Hoare (LH), and Lake Fryxell (LF) basins.


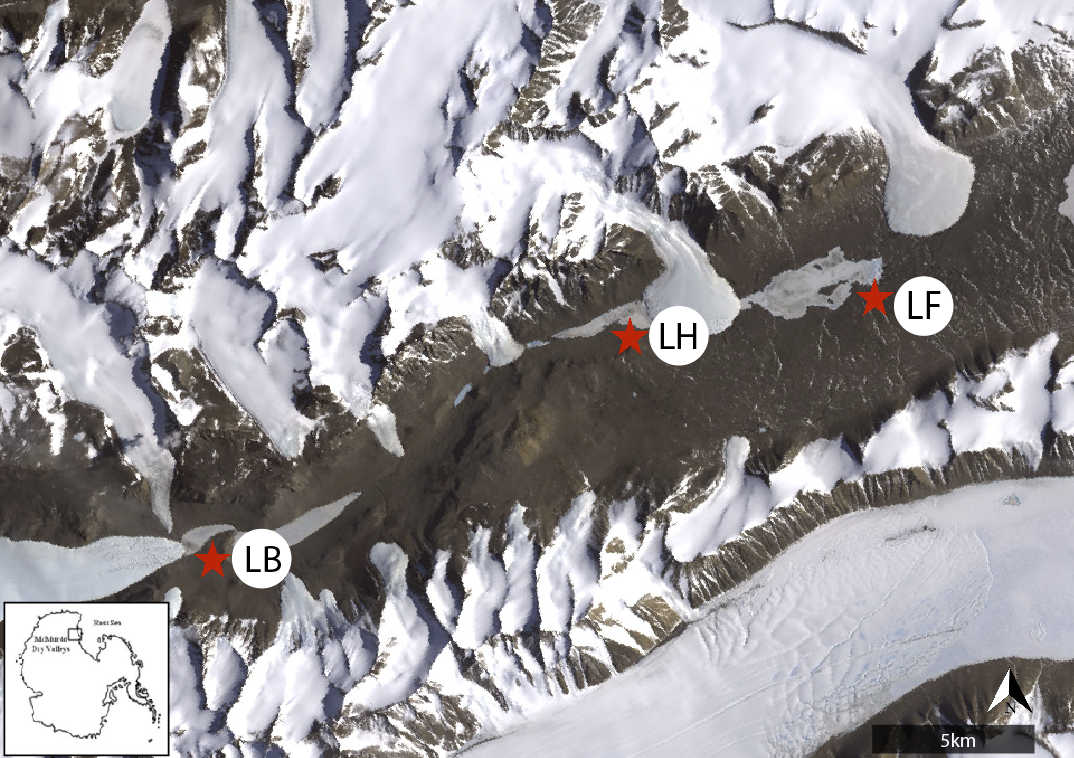


Source: This image was acquired by Landsat 7’s Enhanced Thematic Mapper plus (ETM+) instrument on December 18, 1999. Image by Robert Simmon, based on data provided by the NASA GSFC [Oceans and Ice Branch](http://neptune.gsfc.nasa.gov/oceansandice) and the [Landsat 7 Science Team](http://landsat.gsfc.nasa.gov/). Image retrieved from: <https://earthobservatory.nasa.gov/images/2140/mcmurdo-dry-valleys> on June 6th, 2018.
