# Supplementary figures and images for: Local and Regional Scale Heterogeneity Drive Bacterial Community Diversity and Composition in a Polar Desert
Source: Front Microbiol. 2018 Aug 21;9:1928. doi: 10.3389/fmicb.2018.01928 (PMC6110917; doi:10.3389/fmicb.2018.01928)

Supplemental Figure 2: Relative abundance of major phyla


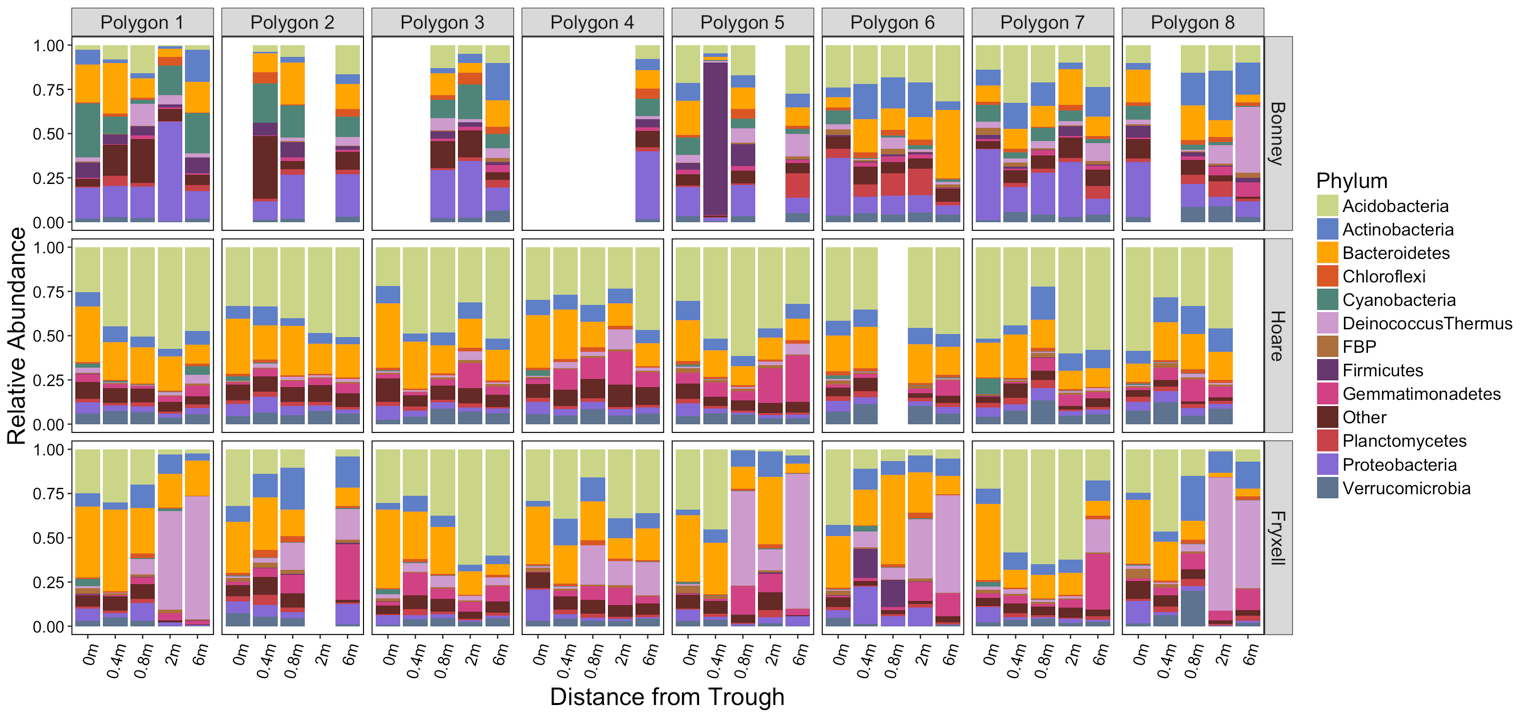

Supplement: Supplementary file 4 [file Data_Sheet_2.docx]

Supplemental Figure 3: Mantel Correlogram using Jaccard distance matrix
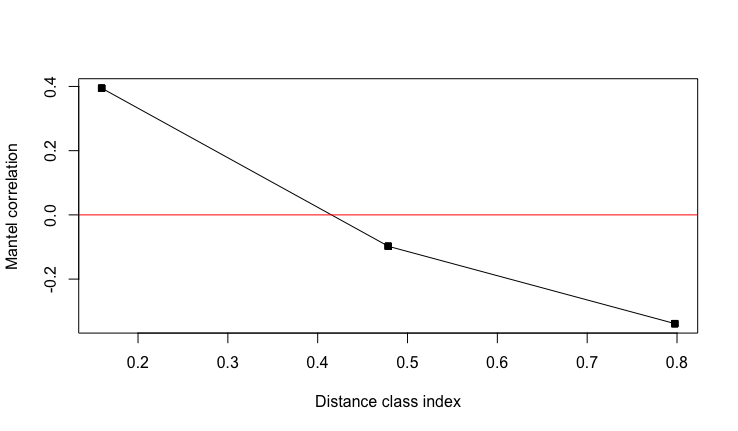

Supplement: Supplementary file 5 [file Data_Sheet_3.docx]
